# Actomyosin stress fiber mechanosensing in 2D and 3D

**DOI:** 10.12688/f1000research.8800.1

**Published:** 2016-09-07

**Authors:** Stacey Lee, Sanjay Kumar

**Affiliations:** 1 Department of Bioengineering, University of California, Berkeley, Berkeley, CA, 94720, USA; 2 UC Berkeley-UCSF Graduate Program in Bioengineering, University of California, Berkeley, Berkeley, CA, 94720, USA; 3 Department of Chemical and Biomolecular Engineering, University of California, Berkeley, Berkeley, CA, 94720, USA

**Keywords:** Myosin, mechanosensing, mechanotransduction, migration, 3D, stress fiber, actomyosin bundle

## Abstract

Mechanotransduction is the process through which cells survey the mechanical properties of their environment, convert these mechanical inputs into biochemical signals, and modulate their phenotype in response. These mechanical inputs, which may be encoded in the form of extracellular matrix stiffness, dimensionality, and adhesion, all strongly influence cell morphology, migration, and fate decisions. One mechanism through which cells on planar or pseudo-planar matrices exert tensile forces and interrogate microenvironmental mechanics is through stress fibers, which are bundles composed of actin filaments and, in most cases, non-muscle myosin II filaments. Stress fibers form a continuous structural network that is mechanically coupled to the extracellular matrix through focal adhesions. Furthermore, myosin-driven contractility plays a central role in the ability of stress fibers to sense matrix mechanics and generate tension. Here, we review the distinct roles that non-muscle myosin II plays in driving mechanosensing and focus specifically on motility. In a closely related discussion, we also describe stress fiber classification schemes and the differing roles of various myosin isoforms in each category. Finally, we briefly highlight recent studies exploring mechanosensing in three-dimensional environments, in which matrix content, structure, and mechanics are often tightly interrelated. Stress fibers and the myosin motors therein represent an intriguing and functionally important biological system in which mechanics, biochemistry, and architecture all converge.

## Introduction

The extracellular matrix (ECM) is a critical regulator of cell and tissue function. Properties of the ECM, including stiffness, topography, and ligand type and density, have all been shown to regulate cell shape, migration, and fate ^1, 2^. For example, matrix stiffness influences the differentiation of mesenchymal and neural stem cells into different lineages ^3– 5^. Substrate topography and stiffness can both direct cell migration and growth ^6– 8^. To effectively probe the properties of the ECM, the cell exerts forces on the environment and gauges the response in a controlled feedback loop that is broadly termed “mechanosensing”.

The cell has specialized machinery for ECM mechanosensing, including motor proteins, cytoskeletal proteins, and force-sensitive proteins that change conformation or activity (or both) in response to applied forces at focal adhesions (FAs), which are protein complexes that directly bind to ECM proteins through integrins and other ECM adhesion receptors ^9– 11^. In one important mode of mechanosensing, the cell uses stress fibers (SFs), which are bundles of 10 to 30 actin filaments in width ^12^ (although some thicker SFs may contain up to ten times as many filaments in width) cross-linked by proteins, including α-actinin. Some SFs also contain non-muscle myosin II (hereafter referred to as MII), which lends contractile properties to the SF and enables the cell to survey ECM physical properties, define cell shape, and facilitate migration. This review will focus on recent advances in SF-based mechanosensing in both two-dimensional (2D) and three-dimensional (3D) environments.

## Myosin structure and regulation

**Figure 1.**
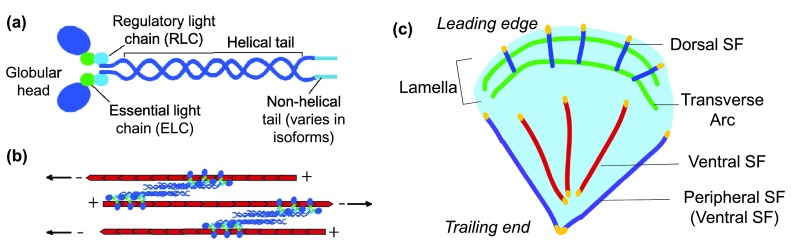
Actomyosin stress fibers in cells. ( **a**) Myosin is composed of two heavy chains, each consisting of a globular head and a tail, two essential light chains, and two regulatory light chains. The non-helical tail region varies in the three isoforms. ( **b**) Myosin heads bind to actin filaments. ATP hydrolysis leads to a conformational change in the head and neck region, which results in mechanical movement of the myosin head toward the plus end of actin and in movement of the actin filament in the opposite direction (indicated by arrows). ( **c**) Stress fibers can be divided into three populations as defined by their anteroposterior position within a migrating cell and connection to focal adhesions. SF, stress fiber.

MII has two important roles in SFs: (1) cross-linking antiparallel actin filaments and (2) generating the power stroke to translocate these filaments to contract the SF. MII is a hexameric protein complex composed of two myosin heavy chains, two essential light chains, and two regulatory light chains (RLCs) ( Figure 1a). The heavy chains contain a helical tail domain and a globular head domain, which can bind to actin filaments and ATP ^13^. Myosin complexes can further organize into bipolar filaments, with the tails in an antiparallel orientation and the actin-bound heads in opposing directions ( Figure 1b). Polarized actin filaments are composed of actin monomers, which are polymerized onto the barbed (plus) end of an existing filament. To contract the filament, myosin heads hydrolyze ATP to generate rotation of the myosin head toward the plus end of actin, leading to the subsequent translocation of antiparallel actin filaments ^14^.

Actomyosin contractility is strongly regulated by phosphorylation of the RLC at Ser19 and Thr18. Ca ^2+^-activated myosin light chain kinase (MLCK) and zipper-interacting protein kinase both phosphorylate the RLCs ^15– 18^. Additionally, Rho-GTPase effectors, including RhoA-activated Rho-associated kinase (ROCK) and p21-associated kinase (PAK), phosphorylate the RLCs ^14, 18, 19^. ROCK can also reduce RLC dephosphorylation via inhibition of myosin light chain phosphatase activity ^14^. Phosphorylation of Ser19 leads to an increase in Mg ^2+^-ATPase activity that powers the MII head sliding along actin filaments and FA maturation ^15, 17, 20^. Additional phosphorylation at Thr18 increases this activity and results in the clustering of actomyosin filaments into thick SFs ^16, 20, 21^. The differential mechanical consequences of mono- versus di-phosphorylation remain an area of active study.

Three myosin isoforms—MIIA, MIIB, and MIIC—have been identified in mammalian cells, differing in their heavy chains. Expression of the three isoforms is not universal in cells. MIIA and MIIB are the predominant isoforms expressed in cultured cells, whereas MIIC is found in a more restricted subset of cells, including neural cells and breast and lung cancer cells ^14, 22^. In recently spread cells that have not yet established polarity, MIIA and MIIB uniformly co-assemble on the same SF ^23, 24^. Over time, as the cell becomes increasingly polarized, the leading edge becomes enriched in MIIA and the trailing end in MIIB ^25– 27^. Although SFs throughout the cell typically contain both MII isoforms, the ratio of MIIA to MIIB is higher in SFs near the leading edge, but decreases as SFs undergo retrograde flow during cytoskeletal remodeling ^23, 28, 29^. This is likely due to a sorting mechanism driven by the different kinetics and heavy chains of the isoforms ^23^. MIIA has a higher turnover rate and spends less time bound to actin compared with MIIB ^30, 31^. As SFs move in a retrograde manner, a higher proportion of MIIA unbinds from the fiber, which in turn enriches the SF in MIIB. Myosin chimeras consisting of swapped C-terminal tails reversed the localization of the isoforms ^32^. These findings are consistent with the presumed differential functions of MIIA and MIIB. Rac1 promotes leading edge formation by generating a flat lamella and recruiting MIIA to the leading edge, where it quickly hydrolyzes ATP to form new, short-lived SFs ^30, 33^. MIIA also stabilizes adhesions and facilitates traction force generation at the leading edge ^34^. On the other hand, MIIB has a slower ATP hydrolysis rate but a higher duty ratio, meaning that it spends more time bound to actin in its force-generating state, thereby generating higher force per ATP hydrolyzed ^31^. This is important in stabilizing SFs, generating traction forces at the trailing edge, and maintaining the front-back polarity needed for directed migration ^23– 26, 35, 36^. Furthermore, MIIB is enriched in perinuclear SFs, where it compresses the cell nucleus to enable efficient cell migration and invasion through confined spaces ^37, 38^. MIIC is less well characterized; it is present in tumor cells and neural cells where it contributes to cytokinesis and neurite growth, respectively ^22, 39, 40^.

## Formation of contractile actomyosin bundles

To determine the minimal requirements for forming SFs, some have employed well-defined reconstituted systems consisting of purified filamentous actin and myosin to study the organization of actin and myosin into contractile bundles. Protein-level cues, including myosin concentration and actin polarity, guide the self-assembly and organization of myosin and actin filaments into contractile bundles, which are the building blocks of the tensed, interconnected SF network ^41– 44^. Analogous to the actomyosin bundles of differing actin polarities that form in reconstituted systems, SFs that vary in actin polarity have been observed in mammalian cells. Three populations of SFs—uniform polarity, graded polarity, and alternating polarity bundles, correlating with the intracellular location of the bundles—were first documented in migrating primary chick fibroblasts ^12^. Uniform polarity bundles were observed near the cell front, and alternating polarity bundles were observed at the cell rear. Graded polarity bundles, in which the degree of polarity depended on the distance from the bundle ends, were located in the center of the cell ^12^.

Recently, careful observation of SF dynamics in migrating U2OS osteosarcoma cells has given rise to a more general classification system for SFs on the basis of their different formation pathways, molecular composition, and connection to FAs ( Figure 1c) ^45, 46^. Dorsal SFs are found at the lamella and have uniform actin polarity, due to inverted formin 2 or vasodilator-stimulated phosphoprotein (VASP) (or both) promoting actin polymerization at the barbed end (closest to the FA) of dorsal SFs ^45– 48^. Furthermore, they are often found to lack MII, implying that dorsal SFs are not contractile ^28, 46, 49^. This subpopulation is connected at one end to an FA, and the other end rises toward the dorsal membrane surface. Dorsal SFs are mechanically coupled to the second subpopulation, transverse arcs. Transverse arcs are curved SFs exhibiting alternating actin polarity, and are found near the dorsal membrane surface of the lamella ^45^. They are formed by the end-to-end annealing of Arp2/3-nucleated actin filaments and are not connected directly to FAs ^46, 50^. Transverse arc contraction, largely driven by MIIA activity, exerts a force on dorsal SFs in the retrograde direction. As dorsal SFs are anchored to the ECM via a stable FA, transverse arc contraction pulls dorsal SFs and the lamella membrane down ^28^. The third subpopulation, ventral SFs, run along the matrix-bound face of the cell, become increasingly prominent toward the cell rear, and are connected at both ends to FAs. A subset of ventral SFs is produced from the myosin-mediated fusion of a transverse arc with two dorsal SFs ^46, 48^. Yet another classification system for SFs distinguishes between peripherally located SFs and centrally located SFs ^29, 51– 54^. This scheme is motivated in part by the recognition that peripheral SFs (sometimes called peripheral arcs) can drive or reflect cortical surface tension (or do both) and that peripheral and central SFs can bear different mechanical loads ^54– 56^.

The primary chick fibroblast SF classification system can perhaps be reconciled with the U2OS SF classification system. The uniform polarity bundles and alternating polarity bundles correspond to dorsal and ventral SFs, respectively. The graded polarity bundles correspond to the transverse arcs fusing with dorsal SFs on either side during retrograde flow ^46^. The degree of polarity corresponds to the location of the SF within a migrating cell. At the lamella, SFs undergoing active and directed polymerization have uniform polarity in order to stabilize the protrusion of the leading edge. As the SFs move toward the trailing edge of the cell, SFs adopt an alternating polarity, indicating that their primary role is to generate contractile forces to maintain cell shape and traction. Peripheral SFs can be classified as ventral SFs (or multiple ventral SFs bundled together), and central SFs can broadly encompass dorsal SFs, transverse arcs, and ventral SFs.

It is important to note that the dorsal/transverse arc/ventral SF was originally developed for mesenchymally migrating cells and that the uniform/graded/alternating polarity system was based on observations in primary chick fibroblasts. The peripheral/central SF classification scheme is the most general and is applicable to many cells. Not all cell types exhibit the dorsal/transverse arc/ventral SF subpopulations, and even within the same population of cells, there may be variability in the representation of each of the SF subpopulations ^28, 45, 46^. Stationary cells often exhibit only ventral SFs, indicating that one of the primary roles of dorsal SFs and transverse arcs is to drive leading edge protrusion during migration. The varying degrees of SF representation raises the question of how different ECM cues, including stiffness, ligand presentation, and dimensionality, collectively influence SF subpopulation formation and organization. Furthermore, there are other questions pertaining to the how SF subpopulations interact to form an interconnected network. For example, transverse arc-dorsal SF junctions are not well characterized at the molecular scale but are likely enriched in actin cross-linking proteins that promote force transmission by tightly coupling dorsal SFs to transverse arcs. These areas are currently under active investigation.

## Stress fiber-based mechanosensing

It is widely appreciated that MII tenses SFs to different degrees in cells. Measurements of the tensile properties of actomyosin bundles have been carried out on reconstituted actomyosin systems or isolated SF networks where all other cell components are removed ^57^. In these simplified systems, SFs can be manipulated to measure their biophysical properties by using tools, including microcantilevers ^58^. However, these methods are not amenable to live cells. Thus, to study SFs in live cells, some have used outside-in perturbations to measure mechanical properties of SFs, including nanoindentation and whole-cell stretching ^59, 60^. Others have used inside-out methods such as pharmacological treatment or genetic perturbations to manipulate SF architecture and tension and measure the resulting changes in the ability of the cell to exert traction on the ECM ^50, 51, 61, 62^. However, with these methods, it is not possible to tease out the mechanical contributions of individual SFs and to examine how they contribute to the overall contractility of the cell.

Thus, our group ^29, 52– 54^ and others ^63^ have used femtosecond laser nanosurgery to sever single SFs to directly measure the mechanical properties, including contractility, of SFs within living cells and confirmed the presumed cross-linking and contractility roles of MII. When ventral SFs are severed, the cut ends retract in a viscoelastic manner which is largely mediated by MII ^52, 53^. MII cross-linking imparts viscous resistance to retraction of a severed SF, as deletion of the actin-binding myosin head speeds SF retraction ^29^. At the same time, MII activity contributes to SF elasticity by tensing actin filaments. The retraction kinetics of SFs differ based on the location of the SF: peripheral SFs retract a longer distance and with a lower effective elasticity (longer time constant) than centrally located SFs, indicating that peripheral SFs are tensed to a greater degree ^53^. These differences may be associated with the spatially compartmentalized control of myosin RLC kinases. Peripheral SFs are preferentially regulated by MLCK, and central SFs by ROCK, as pharmacological inhibition of the kinases using ML-7 (MLCK) or Y-27623 (ROCK) affected the retraction kinetics and morphology of the respective populations ^51, 64^. Some studies suggest that the ratio of MIIA to MIIB isoforms on a particular SF can affect its mechanical properties and that ROCK preferentially regulates MIIA activity whereas MLCK preferentially regulates MIIB ^29, 32, 65^. These findings may be placed in the context of the different mechanochemical properties of MIIA and MIIB. In particular, ROCK-controlled SFs may be enriched in fast ATP-hydrolyzing MIIA, which facilitates the rapid and dynamic SF contraction and evolution in the lamella. MLCK-controlled peripheral SFs may be enriched in high-duty ratio MIIB to support the stable SFs found at stable cell edges. However, additional studies are needed to test these hypothetical associations in a clear and direct way and to examine the differential mechanics of the various SF subpopulations. It would be particularly interesting and important to relate the changes in SF composition and regulation in specific cellular compartments to mechanical functions.

### Traction force generation by stress fibers

MII plays a critical role in sensing mechanical properties of the ECM, including stiffness, by exerting traction stresses on the substrate ^1, 9, 66, 67^. On softer substrates, FAs are smaller and SFs are less abundant as cells are unable to generate sufficient traction that would otherwise reinforce adhesions ^66, 68, 69^. The diminished traction forces can restrict cell spreading and migration, and in some cases are associated with reduced proliferation ^7, 8, 68, 69^. In contrast, cells are able to generate large traction stresses on stiff substrates, which enable them to spread and form mature FAs ^66, 67^. The differences in morphology between cells cultured on compliant and non-compliant substrates are understood to be MII-mediated, because cells lose their characteristic stiffness-dependent differences with abrogation of myosin-based contractility ^8, 70^. Although this review focuses on MII, there are also several other classes of myosin motors whose roles in mechanosensing are under investigation. These myosin motors typically bridge actin filaments to other proteins. For example, myosin X, which links actin to membrane proteins, is critical in the formation of filopodia, thin actin protrusions that participate in ECM remodeling ^71– 73^. In turn, filopodia may contribute to the formation of dorsal SFs ^74^. Future experiments should uncover the roles of other myosin motors in mechanosensing.

Within a given cell, different pools of SFs appear to exert different levels of traction. Although this idea is still being systematically explored, computational analysis of experimental data offers important clues. For example, model-based traction force microscopy (TFM) infers tension held in SFs by iteratively matching traction maps and images of SFs and FAs with cable network models of the actin cytoskeleton ^75^. These measurements reveal that individual ventral SFs exert the highest traction forces and dorsal SFs exert the lowest ^75^. More conventional TFM studies suggest that dorsal SFs are more important for templating the location of adhesions and rely upon MII activity in the cortical actin cytoskeleton (for example, transverse arcs) to drive force-dependent FA growth ^61^. Interestingly, although the traction force per dorsal SF is relatively low, the lamellipodium, which lacks defined SFs, can generate very high traction forces that seem to be largely driven by cortical MII activity ^76, 77^. SF-generated traction likely becomes more important in generating traction forces and defining cell shape in areas further away from the lamellipodium. Dorsal SFs, which are found behind the lamellipodium, directly interact with FAs but can neither generate contractile forces nor exert traction on their own since they lack MII. Instead, they exert low traction forces indirectly through transverse arc contractility. Ventral SFs are the predominant SF type in non-migrating cells, which by definition lack front-back polarity. They are under higher tension and generate higher traction forces than either dorsal SFs or transverse arcs ^75^. Peripherally located ventral SFs collectively exert higher traction stresses compared with centrally located ventral SFs ^52, 54^.

Individual tensed SFs are networked together to form a dynamic system that can readily redistribute tension ^54^. Femtosecond laser nanosurgery is a powerful tool that can be used to obtain mechanical properties of selected SFs and their role in maintaining tension redistribution. For example, a single SF can be severed to elucidate its structural role in the cytoskeleton by examining changes in SF morphology in the surrounding network. Combining this technique with molecular readouts, such as Förster resonance energy transfer (FRET) tension sensors (for example, based on vinculin ^78^, talin ^79^, or α-actinin ^80^), may provide insight into how tension released from a single SF is balanced by the surrounding cytoskeletal network.

### Mechanosensing through a molecular clutch

Mechanosensing by MII pulling on FA-anchored SFs has been described by the motor-clutch model. In this model, MII and FAs respectively act as a cellular motor and clutch mechanism that can probe substrate stiffness and direct actin polymerization ^9^. Spreading cells initiate stiffness sensing by locally tensing the substrate through sarcomeric units consisting of a myosin minifilament (comprised of about 28 myosins arranged in a bipolar fashion) cross-linked to two actin filaments which in turn are connected to nascent focal complexes ^81^. The ECM stiffness value correlates with the number of steps the MII motors take (roughly 2.5 nm per step) before the actin filaments reach a force threshold required to recruit proteins to reinforce and stabilize the adhesion ^81^. Stiff substrates require fewer myosin miniflament steps to recruit and promote nascent adhesions into stable FAs ^81^. These adhesions then associate with SFs and are integrated into the cytoskeletal network, which results in increased tension on the adhesion. These forces unfold mechanically sensitive FA proteins (including talin and vinculin) and, in a positive feedback loop, initiate signaling cascades that produce thicker and highly tensile SFs ^10, 82– 84^. During this process, frictional slippage occurs, whereby actin moves relative to the stationary FA ^9^. Conversely, on more compliant substrates, the myosin miniflaments within a sarcomeric unit are required to take a larger number of steps to reach a force threshold. Load-and-fail dynamics, where the ECM-coupled nascent adhesion moves with the actin filament until a failure point is reached and the adhesion detaches from the ECM, may also be observed ^9, 11, 85– 88^. In this regime, the rate of integrin disengagement from fibronectin, an ECM ligand, is faster than the rate of talin unfolding, which precludes vinculin binding and FA reinforcement ^10^. This results in cells with thinner SFs (or no SFs at all) and cells with smaller projected areas ^68, 89, 90^.

The cytoskeleton undergoes continuous remodeling in response to changes in the environment. When an SF is under tension, VASP is phosphorylated along the SF, leading to increased contractility and a cessation of actin polymerization at the FAs ^48^. It is conceivable that a sarcomeric force-sensing mechanism similar to the one described above at the cell-ECM interface also exists along the length of SFs. That is, on stiff substrates, MII minifilaments would need to take a small number of steps along filaments to reach a threshold force. Increasingly stiff substrates favor the addition of sarcomeric units along the length of the fiber, which incrementally lengthens the SF. This suggests that longer SFs with more sarcomeric units bear more tension. On the other hand, when an SF is no longer under a threshold tension, VASP is not phosphorylated and the SF is targeted for disassembly by cofilin ^48^. These two mechanosensitive mechanisms provide a mechanism for stiffness sensing and durotaxis at the FA and SF: nascent adhesions or SFs that are not under a threshold tension are disassembled, leaving behind stable SFs and adhesions.

MII activity and the ability to sense stiffness cues in the environment mediate various aspects of tumor progression, including dysplasia, tissue invasion, and metastasis ^19^. When manipulated in culture, matrix stiffness and ligand density both affect the ability of cells to migrate, and migration speed is maximized at intermediate levels of both ^91^. These biphasic relationships have been successfully explained by using models that involve myosin-based mechanosensing ^92, 93^. Furthermore, the orientation of matrix proteins, including collagen and fibronectin, determines the ability of cells to effectively engage with the ECM during mechanosensing ^94– 96^. Aberrant mechanosensing has been implicated in the pathogenesis of diseases involving cell migration through tissue, including the invasive brain tumor glioblastoma (GBM). Whereas soft matrices reduce the migration of GBM cell lines in a MII-dependent fashion ^8^, primary GBM tumor-initiating cells spread, migrate, and proliferate even on very compliant matrices ^97^. Increasing myosin contractility through pharmacological or genetic manipulation restores the expected loss of motility, spreading, and proliferation on compliant substrates and dramatically reduces invasion *in vivo* ^97^. Interestingly, myosin activation has also been observed to facilitate GBM cell translocation through tight intercellular spaces within the brain ^37^. Future studies should uncover how the reported *in vitro* roles of MII can be translated into disease microenvironments.

## Actomyosin contractility in three dimensions

Most studies of myosin-mediated SF regulation of cell shape have been conducted in cells cultured on idealized 2D substrates with the basal side interacting with the ECM-coated surface and the dorsal side free. Many of these studies highlighted above focused on one aspect of the microenvironment, such as adhesivity or stiffness, whereas the *in vivo* microenvironment, which is often 3D, can vary in pore/mesh size, degradability, geometry, stiffness, and protein composition. Recent efforts have focused on better understanding the role of complex matrices that are more representative of the *in vivo* conditions, such as interfacial 2D and fully 3D environments. For example, during invasive migration along a blood vessel-ECM interface, tumor cells interact with blood vessels on the basal surface and ECM proteins on their dorsal surface ^98, 99^. In fully 3D environments, cells are often embedded in a meshwork of ECM proteins (for example, collagen) and may interact with several fibers in different planes. The additional dimension introduces another degree of freedom that can significantly alter migration and cell shape from a slowly migrating, lamellipodial shape to a fast migrating, elongated shape ^100^. The role and existence of SFs *in vivo* have been controversial, as they are sometimes assumed to be an artifact of 2D culture ^101, 102^. However, recent publications indicate that contractile SFs are important *in vivo* in processes as varied as wound closure, embryonic epithelial sheet closure, and duct contraction ^103^. Cells also form SFs in 3D matrices consisting of thick collagen bundles ^62, 104^. It is unclear whether 3D SFs, which are often thinner and more difficult to visualize using conventional confocal microscopy techniques, can also be described by the dorsal/transverse arc/ventral SF classification scheme for 2D cultures. Super-resolution imaging and femtosecond laser ablation may be used to better understand the structure, composition, and mechanical properties of these 3D SFs.

### Engineered microenvironments to study mechanotransduction in three-dimensions

To better understand the roles of actomyosin contractility and migration in complex systems and to compare the roles in 2D environments, researchers have used different culture systems to replicate *in vivo* conditions. To mimic interfacial migration, we ^98^ and others ^105^ have developed 2.5D sandwich systems that confine cells between a planar base substrate and an ECM or hydrogel layer. In these systems, cell migration is slower and morphology becomes elongated with no lamellipodia, in the case of GBM cells ^98^. This is attributed to the ECM overlay, which promotes the formation of additional adhesions on the dorsal surface. MII inhibition prevents the formation of strong adhesions to both surfaces and thus enables the cell to migrate faster ^98^.

**Figure 2.**
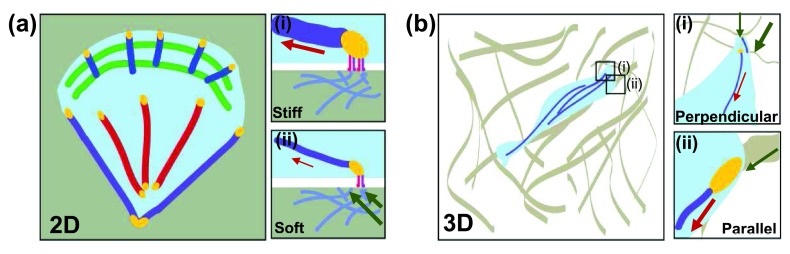
Stress fiber (SF) architecture and cell morphology differ in two-dimensional and three-dimensional matrices. ( **a**) Migrating cells on two-dimensional matrices have a broad, flat leading edge and a pointed trailing end. Dorsal SFs are in blue, transverse arcs in green, ventral SFs in red, and peripheral SFs in purple. Focal adhesions are in yellow. (i) Stiff substrates are able to resist deformation by cell-generated forces (red arrow). This results in focal adhesion maturation and reinforcement of SFs. (ii) Soft substrates deform (green arrows) under cell-generated forces and move with the applied force. Focal adhesions are smaller and SFs are thinner. ( **b**) In three-dimensional collagen matrices, cells adopt an elongated morphology. Collagen fibers have high tensile strength but low resistance to bending. (i) Fibers oriented normally to the cell-generated force (red arrow) readily deform (green arrows). (ii) Fibers oriented parallel to the applied force (red arrow) are tensed (green arrow) and support the formation of mature adhesions and SFs.

Others have also embedded cells in collagen matrices to mimic 3D ECM environments. At the macroscale, collagen forms a soft gel with a 1 kPa Young’s modulus, which is very different from the microscale structure consisting of long fibers with megapascal-scale Young’s modulus (measured from the long axis) that single cells effectively sense ^104^. As in the sandwich cultures, cells adopt an elongated spindle morphology in these matrices and align their adhesions and SFs along collagen fibers ^94, 98, 104^. The local fiber architecture is critical in determining adhesion size and ultimately the magnitude of traction forces that the cell can exert ( Figure 2) ^94^. Collagen has a megapascal-scale tensile strength along the long axis but a much smaller stiffness if measured in the normal direction. Thus, FA area is larger if cell-generated forces are applied parallel to the fiber and smaller if applied normally ^94, 106^.

Given that collagen microarchitecture varies in stiffness and pore size, we ^93, 108^ and others ^38, 100, 109^ have engineered well-defined matrices to decouple these two parameters. We developed a polyacrylamide microchannel platform in which substrate stiffness and confinement can be independently varied. In these environments, cells in stiff, thin-width microchannels form SFs along the channel walls and migrate faster compared with those in soft, wider channels ^93, 107, 108^. The effects of stiff, thin-width channels on cell morphology and migration speed are consistent with those observed in collagen matrices. Others have also fabricated microfluidic devices featuring constrictions of varying widths and found that MIIB is responsible for squeezing the rigid nucleus through these environments ^38, 109^. In yet another approach, thin-width, high-aspect ratio patterned fibronectin strips were used to examine the effects of topography and ligand density on migration and morphology. These “1D” photopatterned strips are reminiscent of the thin fibrillar collagen tracks that cells migrate along in the 3D collagen matrices. In contrast to cells on 2D substrates, cells cultured on the 1D systems have elongated spindle morphologies and fast migration speeds similar to those observed in fibrillar 3D matrices ^100^. Furthermore, unlike in 2D, where the correlation between migration speed and ligand density is biphasic, migration speed is independent of ligand density in 1D ^100^.

Efforts to understand the differential roles of MII isoforms in non-2D systems have also yielded surprising results. MIIA is required to stabilize adhesions and form a flat lamella in 2D and is also required for FA maturation at the leading edge of cells in 1D photopatterned ECMs ^28, 34, 110^. MIIB is required to stabilize mature adhesions further back from the leading edge ^25, 27, 110^. However, inhibition of MII activity has different effects on cells in 2D and 3D. In 2D, genetic ablation of MIIA or pharmacological inhibition of MII activity increases mesenchymal migration speeds, whereas in 3D, migration is abrogated ^34, 104, 110, 111^. This effect is likely due to differences in migration modes: 2D mesenchymal migration is a slow process that is dependent on the formation and maturation of adhesions. Inhibition of MII increases migration speeds by increasing FA turnover and preventing their maturation, which impedes efficient migration ^98^. There is also a possibility that MII inhibition increases the actin monomer pool (which would otherwise be incorporated into thick SFs) at the leading edge, allowing actin polymerization to promote leading edge protrusion for migration. In contrast, in 3D, MII inhibition effectively abrogates migration since actomyosin contractility is needed to break the high levels of integrin clustering that are found in 3D matrices ^104^. Migration by actin polymerization-driven leading edge protrusion is limited since the discontinuous fibers are much smaller in area compared with the 2D case.

## Outlook

*In vivo*, the ECM is highly complex and variable in stiffness, dimensionality, and ligand presentation. These different combinations of matrix properties may influence cell behavior in complex and unpredictable ways that are challenging to deduce from studies in which single properties are varied in isolation. Although it seems clear that MII-mediated actomyosin contractility within SFs plays crucial roles in mechanosensing in 2D culture, the field is still grappling with the translation of these relationships to more complex microenvironments representative of tissue. Thus, an important objective going forward will be to characterize these relationships, which will surely be facilitated by developing more sophisticated culture paradigms.

## References

[1] Discher DE Janmey P Wang YL: Tissue cells feel and respond to the stiffness of their substrate. *Science.* 2005;310(5751):1139–43. 10.1126/science.1116995 16293750

[2] Engler A Bacakova L Newman C: Substrate compliance versus ligand density in cell on gel responses. *Biophys J.* 2004;86(1 Pt 1):617–28. 10.1016/S0006-3495(04)74140-5 14695306 PMC1303831

[3] Engler AJ Sen S Sweeney HL: Matrix elasticity directs stem cell lineage specification. *Cell.* 2006;126(4):677–89. 10.1016/j.cell.2006.06.044 16923388

[4] Keung AJ Asuri P Kumar S: Soft microenvironments promote the early neurogenic differentiation but not self-renewal of human pluripotent stem cells. *Integr Biol (Camb).* 2012;4(9):1049–58. 10.1039/c2ib20083j 22854634 PMC3459311

[5] Keung AJ de Juan-Pardo EM Schaffer DV: Rho GTPases mediate the mechanosensitive lineage commitment of neural stem cells. *Stem Cells.* 2011;29(11):1886–97. 10.1002/stem.746 21956892 PMC5990277

[6] Rajnicek A Britland S McCaig C: Contact guidance of CNS neurites on grooved quartz: influence of groove dimensions, neuronal age and cell type. *J Cell Sci.* 1997;110(Pt 23):2905–13. 9359873 10.1242/jcs.110.23.2905

[7] Lo CM Wang HB Dembo M: Cell movement is guided by the rigidity of the substrate. *Biophys J.* 2000;79(1):144–52. 10.1016/S0006-3495(00)76279-5 10866943 PMC1300921

[8] Ulrich TA de Juan Pardo EM Kumar S: The mechanical rigidity of the extracellular matrix regulates the structure, motility, and proliferation of glioma cells. *Cancer Res.* 2009;69(10):4167–74. 10.1158/0008-5472.CAN-08-4859 19435897 PMC2727355

[9] Chan CE Odde DJ: Traction dynamics of filopodia on compliant substrates. *Science.* 2008;322(5908):1687–91. 10.1126/science.1163595 19074349

[10] Elosegui-Artola A Oria R Chen Y: Mechanical regulation of a molecular clutch defines force transmission and transduction in response to matrix rigidity. *Nat Cell Biol.* 2016;18(5):540–8. 10.1038/ncb3336 27065098

[11] Aratyn-Schaus Y Gardel ML: Transient frictional slip between integrin and the ECM in focal adhesions under myosin II tension. *Curr Biol.* 2010;20(13):1145–53. 10.1016/j.cub.2010.05.049 20541412 PMC2902720

[12] Cramer LP Siebert M Mitchison TJ: Identification of novel graded polarity actin filament bundles in locomoting heart fibroblasts: implications for the generation of motile force. *J Cell Biol.* 1997;136(6):1287–305. 10.1083/jcb.136.6.1287 9087444 PMC2132518

[13] Niederman R Pollard TD: Human platelet myosin. II. *In vitro* assembly and structure of myosin filaments. *J Cell Biol.* 1975;67(1):72–92. 10.1083/jcb.67.1.72 240861 PMC2109578

[14] Vicente-Manzanares M Ma X Adelstein RS: Non-muscle myosin II takes centre stage in cell adhesion and migration. *Nat Rev Mol Cell Biol.* 2009;10(11):778–90. 10.1038/nrm2786 19851336 PMC2834236

[15] Ikebe M Hartshorne DJ: Phosphorylation of smooth muscle myosin at two distinct sites by myosin light chain kinase. *J Biol Chem.* 1985;260(18):10027–31. 3839510

[16] Ikebe M Koretz J Hartshorne DJ: Effects of phosphorylation of light chain residues threonine 18 and serine 19 on the properties and conformation of smooth muscle myosin. *J Biol Chem.* 1988;263(13):6432–7. 2966156

[17] Ikebe M Hartshorne DJ Elzinga M: Phosphorylation of the 20,000-dalton light chain of smooth muscle myosin by the calcium-activated, phospholipid-dependent protein kinase. Phosphorylation sites and effects of phosphorylation. *J Biol Chem.* 1987;262(20):9569–73. 3036866

[18] Murata-Hori M Suizu F Iwasaki T: ZIP kinase identified as a novel myosin regulatory light chain kinase in HeLa cells. *FEBS Lett.* 1999;451(1):81–4. 10.1016/S0014-5793(99)00550-5 10356987

[19] Newell-Litwa KA Horwitz R Lamers ML: Non-muscle myosin II in disease: mechanisms and therapeutic opportunities. *Dis Model Mech.* 2015;8(12):1495–515. 10.1242/dmm.022103 26542704 PMC4728321

[20] Vicente-Manzanares M Horwitz AR: Myosin light chain mono- and di-phosphorylation differentially regulate adhesion and polarity in migrating cells. *Biochem Biophys Res Commun.* 2010;402(3):537–42. 10.1016/j.bbrc.2010.10.071 20971064 PMC2991406

[21] Chrzanowska-Wodnicka M Burridge K: Rho-stimulated contractility drives the formation of stress fibers and focal adhesions. *J Cell Biol.* 1996;133(6):1403–15. 8682874 10.1083/jcb.133.6.1403 PMC2120895

[22] Jana SS Kawamoto S Adelstein RS: A specific isoform of nonmuscle myosin II-C is required for cytokinesis in a tumor cell line. *J Biol Chem.* 2006;281(34):24662–70. 10.1074/jbc.M604606200 16790446

[23] Beach JR Shao L Remmert K: Nonmuscle myosin II isoforms coassemble in living cells. *Curr Biol.* 2014;24(10):1160–6. 10.1016/j.cub.2014.03.071 24814144 PMC4108432

[24] Shutova MS Spessott WA Giraudo CG: Endogenous species of mammalian nonmuscle myosin IIA and IIB include activated monomers and heteropolymers. *Curr Biol.* 2014;24(17):1958–68. 10.1016/j.cub.2014.07.070 25131674 PMC4160463

[25] Vicente-Manzanares M Newell-Litwa K Bachir AI: Myosin IIA/IIB restrict adhesive and protrusive signaling to generate front-back polarity in migrating cells. *J Cell Biol.* 2011;193(2):381–96. 10.1083/jcb.201012159 21482721 PMC3080254

[26] Vicente-Manzanares M Koach MA Whitmore L: Segregation and activation of myosin IIB creates a rear in migrating cells. *J Cell Biol.* 2008;183(3):543–54. 10.1083/jcb.200806030 18955554 PMC2575793

[27] Vicente-Manzanares M Zareno J Whitmore L: Regulation of protrusion, adhesion dynamics, and polarity by myosins IIA and IIB in migrating cells. *J Cell Biol.* 2007;176(5):573–80. 10.1083/jcb.200612043 17312025 PMC2064016

[28] Burnette DT Shao L Ott C: A contractile and counterbalancing adhesion system controls the 3D shape of crawling cells. *J Cell Biol.* 2014;205(1):83–96. 10.1083/jcb.201311104 24711500 PMC3987145

[29] Chang CW Kumar S: Differential Contributions of Nonmuscle Myosin II Isoforms and Functional Domains to Stress Fiber Mechanics. *Sci Rep.* 2015;5:13736. 10.1038/srep13736 26336830 PMC4559901

[30] Kovács M Wang F Hu A: Functional divergence of human cytoplasmic myosin II: kinetic characterization of the non-muscle IIA isoform. *J Biol Chem.* 2003;278(40):38132–40. 10.1074/jbc.M305453200 12847096

[31] Wang F Kovacs M Hu A: Kinetic mechanism of non-muscle myosin IIB: functional adaptations for tension generation and maintenance. *J Biol Chem.* 2003;278(30):27439–48. 10.1074/jbc.M302510200 12704189

[32] Sandquist JC Means AR: The C-terminal tail region of nonmuscle myosin II directs isoform-specific distribution in migrating cells. *Mol Biol Cell.* 2008;19(12):5156–67. 10.1091/mbc.E08-05-0533 18843042 PMC2592670

[33] Pasapera AM Plotnikov SV Fischer RS: Rac1-dependent phosphorylation and focal adhesion recruitment of myosin IIA regulates migration and mechanosensing. *Curr Biol.* 2015;25(2):175–86. 10.1016/j.cub.2014.11.043 25544611 PMC4302036

[34] Jorrisch MH Shih W Yamada S: Myosin IIA deficient cells migrate efficiently despite reduced traction forces at cell periphery. *Biol Open.* 2013;2(4):368–72. 10.1242/bio.20133707 23616920 PMC3625864

[35] Lo CM Buxton DB Chua GC: Nonmuscle myosin IIb is involved in the guidance of fibroblast migration. *Mol Biol Cell.* 2004;15(3):982–9. 10.1091/mbc.E03-06-0359 14699073 PMC363055

[36] Beach JR Hammer JA 3rd: Myosin II isoform co-assembly and differential regulation in mammalian systems. *Exp Cell Res.* 2015;334(1):2–9. 10.1016/j.yexcr.2015.01.012 25655283 PMC4433797

[37] Beadle C Assanah MC Monzo P: The role of myosin II in glioma invasion of the brain. *Mol Biol Cell.* 2008;19(8):3357–68. 10.1091/mbc.E08-03-0319 18495866 PMC2488307

[38] Thomas DG Yenepalli A Denais CM: Non-muscle myosin IIB is critical for nuclear translocation during 3D invasion. *J Cell Biol.* 2015;210(4):583–94. 10.1083/jcb.201502039 26261182 PMC4539979

[39] Takaoka M Saito H Takenaka K: BRCA2 phosphorylated by PLK1 moves to the midbody to regulate cytokinesis mediated by nonmuscle myosin IIC. *Cancer Res.* 2014;74(5):1518–28. 10.1158/0008-5472.CAN-13-0504 24448238

[40] Wylie SR Chantler PD: Myosin IIC: a third molecular motor driving neuronal dynamics. *Mol Biol Cell.* 2008;19(9):3956–68. 10.1091/mbc.E07-08-0744 18614800 PMC2526701

[41] Thoresen T Lenz M Gardel ML: Reconstitution of contractile actomyosin bundles. *Biophys J.* 2011;100(11):2698–705. 10.1016/j.bpj.2011.04.031 21641315 PMC3117186

[42] Stachowiak MR McCall PM Thoresen T: Self-organization of myosin II in reconstituted actomyosin bundles. *Biophys J.* 2012;103(6):1265–74. 10.1016/j.bpj.2012.08.028 22995499 PMC3446672

[43] Murrell MP Gardel ML: F-actin buckling coordinates contractility and severing in a biomimetic actomyosin cortex. *Proc Natl Acad Sci U S A.* 2012;109(51):20820–5. 10.1073/pnas.1214753109 23213249 PMC3529094

[44] Murrell M Gardel ML: Actomyosin sliding is attenuated in contractile biomimetic cortices. *Mol Biol Cell.* 2014;25(12):1845–53. 10.1091/mbc.E13-08-0450 24760970 PMC4055264

[45] Small JV Rottner K Kaverina I: Assembling an actin cytoskeleton for cell attachment and movement. *Biochim Biophys Acta.* 1998;1404(3):271–81. 10.1016/S0167-4889(98)00080-9 9739149

[46] Hotulainen P Lappalainen P: Stress fibers are generated by two distinct actin assembly mechanisms in motile cells. *J Cell Biol.* 2006;173(3):383–94. 10.1083/jcb.200511093 16651381 PMC2063839

[47] Skau CT Plotnikov SV Doyle AD: Inverted formin 2 in focal adhesions promotes dorsal stress fiber and fibrillar adhesion formation to drive extracellular matrix assembly. *Proc Natl Acad Sci U S A.* 2015;112(19):E2447–56. 10.1073/pnas.1505035112 25918420 PMC4434736

[48] Tojkander S Gateva G Husain A: Generation of contractile actomyosin bundles depends on mechanosensitive actin filament assembly and disassembly. *eLife.* 2015;4:e06126. 10.7554/eLife.06126 26652273 PMC4714978

[49] Kovac B Teo JL Mäkelä TP: Assembly of non-contractile dorsal stress fibers requires α-actinin-1 and Rac1 in migrating and spreading cells. *J Cell Sci.* 2013;126(Pt 1):263–73. 10.1242/jcs.115063 23132927

[50] Tojkander S Gateva G Schevzov G: A molecular pathway for myosin II recruitment to stress fibers. *Curr Biol.* 2011;21(7):539–50. 10.1016/j.cub.2011.03.007 21458264

[51] Totsukawa G Yamakita Y Yamashiro S: Distinct roles of ROCK (Rho-kinase) and MLCK in spatial regulation of MLC phosphorylation for assembly of stress fibers and focal adhesions in 3T3 fibroblasts. *J Cell Biol.* 2000;150(4):797–806. 10.1083/jcb.150.4.797 10953004 PMC2175273

[52] Kumar S Maxwell IZ Heisterkamp A: Viscoelastic retraction of single living stress fibers and its impact on cell shape, cytoskeletal organization, and extracellular matrix mechanics. *Biophys J.* 2006;90(10):3762–73. 10.1529/biophysj.105.071506 16500961 PMC1440757

[53] Tanner K Boudreau A Bissell MJ: Dissecting regional variations in stress fiber mechanics in living cells with laser nanosurgery. *Biophys J.* 2010;99(9):2775–83. 10.1016/j.bpj.2010.08.071 21044574 PMC2965957

[54] Chang CW Kumar S: Vinculin tension distributions of individual stress fibers within cell-matrix adhesions. *J Cell Sci.* 2013;126(Pt 14):3021–30. 10.1242/jcs.119032 23687380 PMC3711198

[55] Albert PJ Schwarz US: Dynamics of cell shape and forces on micropatterned substrates predicted by a cellular Potts model. *Biophys J.* 2014;106(11):2340–52. 10.1016/j.bpj.2014.04.036 24896113 PMC4052361

[56] Kumar A Ouyang M Van den Dries K: Talin tension sensor reveals novel features of focal adhesion force transmission and mechanosensitivity. *J Cell Biol.* 2016;213(3):371–83. 10.1083/jcb.201510012 27161398 PMC4862330

[57] Kassianidou E Kumar S: A biomechanical perspective on stress fiber structure and function. *Biochim Biophys Acta.* 2015;1853(11 Pt B):3065–74. 10.1016/j.bbamcr.2015.04.006 25896524 PMC4589434

[58] Deguchi S Ohashi T Sato M: Tensile properties of single stress fibers isolated from cultured vascular smooth muscle cells. *J Biomech.* 2006;39(14):2603–10. 10.1016/j.jbiomech.2005.08.026 16216252

[59] Lu L Oswald SJ Ngu H: Mechanical properties of actin stress fibers in living cells. *Biophys J.* 2008;95(12):6060–71. 10.1529/biophysj.108.133462 18820238 PMC2599828

[60] Costa KD Hucker WJ Yin FC: Buckling of actin stress fibers: a new wrinkle in the cytoskeletal tapestry. *Cell Motil Cytoskeleton.* 2002;52(4):266–74. 10.1002/cm.10056 12112140

[61] Oakes PW Beckham Y Stricker J: Tension is required but not sufficient for focal adhesion maturation without a stress fiber template. *J Cell Biol.* 2012;196(3):363–74. 10.1083/jcb.201107042 22291038 PMC3275371

[62] Gateva G Tojkander S Koho S: Palladin promotes assembly of non-contractile dorsal stress fibers through VASP recruitment. *J Cell Sci.* 2014;127(Pt 9):1887–98. 10.1242/jcs.135780 24496446

[63] Colombelli J Besser A Kress H: Mechanosensing in actin stress fibers revealed by a close correlation between force and protein localization. *J Cell Sci.* 2009;122(Pt 10):1665–79. 10.1242/jcs.042986 19401336

[64] Katoh K Kano Y Amano M: Stress fiber organization regulated by MLCK and Rho-kinase in cultured human fibroblasts. *Am J Physiol Cell Physiol.* 2001;280(6):C1669–79. 11350763 10.1152/ajpcell.2001.280.6.C1669

[65] Sandquist JC Swenson KI Demali KA: Rho kinase differentially regulates phosphorylation of nonmuscle myosin II isoforms A and B during cell rounding and migration. *J Biol Chem.* 2006;281(47):35873–83. 10.1074/jbc.M605343200 17020881

[66] Guo WH Frey MT Burnham NA: Substrate rigidity regulates the formation and maintenance of tissues. *Biophys J.* 2006;90(6):2213–20. 10.1529/biophysj.105.070144 16387786 PMC1386800

[67] Califano JP Reinhart-King CA: Substrate Stiffness and Cell Area Predict Cellular Traction Stresses in Single Cells and Cells in Contact. *Cell Mol Bioeng.* 2010;3(1):68–75. 10.1007/s12195-010-0102-6 21116436 PMC2992361

[68] Galbraith CG Yamada KM Sheetz MP: The relationship between force and focal complex development. *J Cell Biol.* 2002;159(4):695–705. 10.1083/jcb.200204153 12446745 PMC2173098

[69] Pelham RJ Jr Wang Yl: Cell locomotion and focal adhesions are regulated by substrate flexibility. *Proc Natl Acad Sci U S A.* 1997;94(25):13661–5. 10.1073/pnas.94.25.13661 9391082 PMC28362

[70] Mih JD Marinkovic A Liu F: Matrix stiffness reverses the effect of actomyosin tension on cell proliferation. *J Cell Sci.* 2012;125(Pt 24):5974–83. 10.1242/jcs.108886 23097048 PMC3585515

[71] Zhang H Berg JS Li Z: Myosin-X provides a motor-based link between integrins and the cytoskeleton. *Nat Cell Biol.* 2004;6(6):523–31. 10.1038/ncb1136 15156152

[72] Tokuo H Ikebe M: Myosin X transports Mena/VASP to the tip of filopodia. *Biochem Biophys Res Commun.* 2004;319(1):214–20. 10.1016/j.bbrc.2004.04.167 15158464

[73] Bohil AB Robertson BW Cheney RE: Myosin-X is a molecular motor that functions in filopodia formation. *Proc Natl Acad Sci U S A.* 2006;103(33):12411–6. 10.1073/pnas.0602443103 16894163 PMC1567893

[74] Nemethova M Auinger S Small JV: Building the actin cytoskeleton: filopodia contribute to the construction of contractile bundles in the lamella. *J Cell Biol.* 2008;180(6):1233–44. 10.1083/jcb.200709134 18362182 PMC2290848

[75] Soiné JR Brand CA Stricker J: Model-based traction force microscopy reveals differential tension in cellular actin bundles. *PLoS Comput Biol.* 2015;11(3):e1004076. 10.1371/journal.pcbi.1004076 25748431 PMC4352062

[76] Stricker J Aratyn-Schaus Y Oakes PW: Spatiotemporal constraints on the force-dependent growth of focal adhesions. *Biophys J.* 2011;100(12):2883–93. 10.1016/j.bpj.2011.05.023 21689521 PMC3123981

[77] Beningo KA Dembo M Kaverina I: Nascent focal adhesions are responsible for the generation of strong propulsive forces in migrating fibroblasts. *J Cell Biol.* 2001;153(4):881–8. 10.1083/jcb.153.4.881 11352946 PMC2192381

[78] Grashoff C Hoffman BD Brenner MD: Measuring mechanical tension across vinculin reveals regulation of focal adhesion dynamics. *Nature.* 2010;466(7303):263–6. 10.1038/nature09198 20613844 PMC2901888

[79] Austen K Ringer P Mehlich A: Extracellular rigidity sensing by talin isoform-specific mechanical linkages. *Nat Cell Biol.* 2015;17(12):1597–606. 10.1038/ncb3268 26523364 PMC4662888

[80] Meng F Sachs F: Visualizing dynamic cytoplasmic forces with a compliance-matched FRET sensor. *J Cell Sci.* 2011;124(Pt 2):261–9. 10.1242/jcs.071928 21172803 PMC3010192

[81] Wolfenson H Meacci G Liu S: Tropomyosin controls sarcomere-like contractions for rigidity sensing and suppressing growth on soft matrices. *Nat Cell Biol.* 2016;18(1):33–42. 10.1038/ncb3277 26619148 PMC5296190

[82] Jiang G Giannone G Critchley DR: Two-piconewton slip bond between fibronectin and the cytoskeleton depends on talin. *Nature.* 2003;424(6946):334–7. 10.1038/nature01805 12867986

[83] Giannone G Jiang G Sutton DH: Talin1 is critical for force-dependent reinforcement of initial integrin-cytoskeleton bonds but not tyrosine kinase activation. *J Cell Biol.* 2003;163(2):409–19. 10.1083/jcb.200302001 14581461 PMC2173516

[84] del Rio A Perez-Jimenez R Liu R: Stretching single talin rod molecules activates vinculin binding. *Science.* 2009;323(5914):638–41. 10.1126/science.1162912 19179532 PMC9339221

[85] Mitchison T Kirschner M: Cytoskeletal dynamics and nerve growth. *Neuron.* 1988;1(9):761–72. 10.1016/0896-6273(88)90124-9 3078414

[86] Hu K Ji L Applegate KT: Differential transmission of actin motion within focal adhesions. *Science.* 2007;315(5808):111–5. 10.1126/science.1135085 17204653

[87] Smilenov LB Mikhailov A Pelham RJ Jr: Focal adhesion motility revealed in stationary fibroblasts. *Science.* 1999;286(5442):1172–4. 10.1126/science.286.5442.1172 10550057

[88] Jurado C Haserick JR Lee J: Slipping or gripping? Fluorescent speckle microscopy in fish keratocytes reveals two different mechanisms for generating a retrograde flow of actin. *Mol Biol Cell.* 2005;16(2):507–18. 10.1091/mbc.E04-10-0860 15548591 PMC545886

[89] Yeung T Georges PC Flanagan LA: Effects of substrate stiffness on cell morphology, cytoskeletal structure, and adhesion. *Cell Motil Cytoskeleton.* 2005;60(1):24–34. 10.1002/cm.20041 15573414

[90] Choi CK Vicente-Manzanares M Zareno J: Actin and alpha-actinin orchestrate the assembly and maturation of nascent adhesions in a myosin II motor-independent manner. *Nat Cell Biol.* 2008;10(9):1039–50. 10.1038/ncb1763 19160484 PMC2827253

[91] Peyton SR Putnam AJ: Extracellular matrix rigidity governs smooth muscle cell motility in a biphasic fashion. *J Cell Physiol.* 2005;204(1):198–209. 10.1002/jcp.20274 15669099

[92] Pathak A Kumar S: From molecular signal activation to locomotion: an integrated, multiscale analysis of cell motility on defined matrices. *PLoS One.* 2011;6(3):e18423. 10.1371/journal.pone.0018423 21483802 PMC3069105

[93] Pathak A Kumar S: Independent regulation of tumor cell migration by matrix stiffness and confinement. *Proc Natl Acad Sci U S A.* 2012;109(26):10334–9. 10.1073/pnas.1118073109 22689955 PMC3387066

[94] Kubow KE Conrad SK Horwitz AR: Matrix microarchitecture and myosin II determine adhesion in 3D matrices. *Curr Biol.* 2013;23(17):1607–19. 10.1016/j.cub.2013.06.053 23932405 PMC3773288

[95] Garcia AJ Vega MD Boettiger D: Modulation of cell proliferation and differentiation through substrate-dependent changes in fibronectin conformation. *Mol Biol Cell.* 1999;10(3):785–98. 10.1091/mbc.10.3.785 10069818 PMC25202

[96] Lee JP Kassianidou E MacDonald JI: N-terminal specific conjugation of extracellular matrix proteins to 2-pyridinecarboxaldehyde functionalized polyacrylamide hydrogels. *Biomaterials.* 2016;102:268–76. 10.1016/j.biomaterials.2016.06.022 27348850 PMC4939314

[97] Wong SY Ulrich TA Deleyrolle LP: Constitutive activation of myosin-dependent contractility sensitizes glioma tumor-initiating cells to mechanical inputs and reduces tissue invasion. *Cancer Res.* 2015;75(6):1113–22. 10.1158/0008-5472.CAN-13-3426 25634210 PMC4359960

[98] Rape AD Kumar S: A composite hydrogel platform for the dissection of tumor cell migration at tissue interfaces. *Biomaterials.* 2014;35(31):8846–53. 10.1016/j.biomaterials.2014.07.003 25047626 PMC4127155

[99] Friedl P Alexander S: Cancer invasion and the microenvironment: plasticity and reciprocity. *Cell.* 2011;147(5):992–1009. 10.1016/j.cell.2011.11.016 22118458

[100] Doyle AD Wang FW Matsumoto K: One-dimensional topography underlies three-dimensional fibrillar cell migration. *J Cell Biol.* 2009;184(4):481–90. 10.1083/jcb.200810041 19221195 PMC2654121

[101] Burridge K Wittchen ES: The tension mounts: stress fibers as force-generating mechanotransducers. *J Cell Biol.* 2013;200(1):9–19. 10.1083/jcb.201210090 23295347 PMC3542796

[102] Vallenius T: Actin stress fibre subtypes in mesenchymal-migrating cells. *Open Biol.* 2013;3(6):130001. 10.1098/rsob.130001 23782578 PMC3718327

[103] Pellegrin S Mellor H: Actin stress fibres. *J Cell Sci.* 2007;120(Pt 20):3491–9. 10.1242/jcs.018473 17928305

[104] Doyle AD Carvajal N Jin A: Local 3D matrix microenvironment regulates cell migration through spatiotemporal dynamics of contractility-dependent adhesions. *Nat Commun.* 2015;6:8720. 10.1038/ncomms9720 26548801 PMC4643399

[105] Berthiaume F Moghe PV Toner M: Effect of extracellular matrix topology on cell structure, function, and physiological responsiveness: hepatocytes cultured in a sandwich configuration. *FASEB J.* 1996;10(13):1471–84. 8940293 10.1096/fasebj.10.13.8940293

[106] Doyle AD Yamada KM: Mechanosensing via cell-matrix adhesions in 3D microenvironments. *Exp Cell Res.* 2016;343(1):60–6. 10.1016/j.yexcr.2015.10.033 26524505 PMC4851608

[107] Rape A Ananthanarayanan B Kumar S: Engineering strategies to mimic the glioblastoma microenvironment. *Adv Drug Deliv Rev.* 2014;79–80:172–83. 10.1016/j.addr.2014.08.012 25174308 PMC4258440

[108] Pathak A Kumar S: Transforming potential and matrix stiffness co-regulate confinement sensitivity of tumor cell migration. *Integr Biol (Camb).* 2013;5(8):1067–75. 10.1039/c3ib40017d 23832051 PMC3755126

[109] Denais CM Gilbert RM Isermann P: Nuclear envelope rupture and repair during cancer cell migration. *Science.* 2016;352(6283):353–8. 10.1126/science.aad7297 27013428 PMC4833568

[110] Doyle AD Kutys ML Conti MA: Micro-environmental control of cell migration--myosin IIA is required for efficient migration in fibrillar environments through control of cell adhesion dynamics. *J Cell Sci.* 2012;125(Pt 9):2244–56. 10.1242/jcs.098806 22328520 PMC3367941

[111] Shih W Yamada S: Myosin IIA dependent retrograde flow drives 3D cell migration. *Biophys J.* 2010;98(8):L29–31. 10.1016/j.bpj.2010.02.028 20409454 PMC2856159

